# Effect of Body Position on Electrical Activity of Respiratory Muscles During Mouth and Nasal Maximal Respiratory Pressure in Healthy Adults: A Pilot Study

**DOI:** 10.3390/jfmk9040241

**Published:** 2024-11-17

**Authors:** Lailane Saturnino da Silva, Rayane Grayce da Silva Vieira, Thiago Bezerra Wanderley e Lima, Vanessa Regiane Resqueti, Jordi Vilaro, Jessica Danielle Medeiros da Fonseca, Giane Amorim Ribeiro-Samora, Guilherme Augusto de Freitas Fregonezi

**Affiliations:** 1 PneumoCardioVascular Laboratory/HUOL, Hospital Universitário Onofre Lopes, Departamento de Fisioterapia Universidade Federal do Rio Grande do Norte, Natal 59078-970, Brazil; lailane.saturnino.074@ufrn.edu.br (L.S.d.S.); rayane.vieira.071@ufrn.edu.br (R.G.d.S.V.); thiagowanderley13@hotmail.com (T.B.W.e.L.); vanessa.resqueti@ufrn.br (V.R.R.); jessy_nielle@hotmail.com (J.D.M.d.F.); 2 Laboratório de Inovação Tecnológica em Reabilitação, Departamento de Fisioterapia, Universidade Federal do Rio Grande do Norte, Natal 59078-970, Brazil; 3 Blanquerna Faculty of Health Sciences, GRoW—Global Research on Wellbeing, Ramon Llull University, 08022 Barcelona, Spain; jordivc@blanquerna.url.edu; 4 Departamento de Fisioterapia, Universidade Federal do Rio Grande do Norte, Natal 59078-970, Brazil; giane.samora@ufrn.br

**Keywords:** respiratory muscles, maximum respiratory pressures, electromyography, posture

## Abstract

**Background**: This study aimed to analyze the impact of seated, 45° inclined, and supine positions on respiratory muscle strength (Maximal Inspiratory Pressure—MIP, Maximal Expiratory Pressure—MEP, Sniff Nasal Inspiratory Pressure—SNIP and Sniff Nasal Expiratory Pressure—SNEP) and the electrical activity of respiratory muscles in healthy adults. Ten healthy subjects were evaluated. **Methods**: Personal, anthropometric data (weight, height, BMI) and lung function (spirometry) were collected, followed by random assessments of inspiratory (MIP, SNIP) and expiratory (MEP, SNEP) muscle strength. Respiratory muscle strength maneuvers and surface electromyographic (sEMG) activity were assessed in sitting, 45° inclined, and supine positions. **Results**: present that MIP was statistically higher in the sitting position compared to the supine position (*p* < 0.05) and the 45° supine position (*p* < 0.05), with SNIP: *p* < 0.05 and SNEP: *p* < 0.05 as well. Intercostal muscle activity was higher during MIP, MEP, and SNEP maneuvers in the sitting position (*p* < 0.05). Additionally, rectus abdominis muscle activity was higher in this position during MIP and SNEP maneuvers. **Conclusions**: The results suggest there are significant differences in inspiratory pressures between positions, with the difference in activity muscle pattern. In conclusion, body position affected maximal respiratory pressures and influences EMG activation of specific respiratory muscles during MIP.

## 1. Introduction

The ability of the neuromuscular system to generate power is affected by a few factors, including the length–tension relationship. Additionally, maximum power production is influenced by morphological factors, muscular architectural characteristics, as well as neural factors, including synchronization and intermuscular coordination [1]. However, research conducted over a couple of decades has not detailed the mechanics of how respiratory muscle might be influenced by body position. While some studies have suggested no correlation between body position and respiratory muscle strength in healthy people [2], others have suggested a correlation [3]. Still others have found that people with chronic respiratory disease have inconsistent results in terms of respiratory muscle strength [4,5,6].

The influence of body position on ventilation has been studied for several decades, both in healthy people [2] and in patients with a wide range of respiratory conditions [7,8]. Changes in posture have the potential to alter the length and position of the respiratory muscles, thereby affecting their function, which in turn affects their ability to generate tension and, consequently, their ability to generate instantaneous force [1].

Several techniques are available to assess respiratory muscle strength using different measurement protocols. These include the assessment of maximum inspiratory and expiratory pressures at the mouth (Maximal Inspiratory Pressure—MIP and Maximal Expiratory Pressure—MEP) [9], and sniff nasal inspiratory and expiratory pressures (SNIP and SNEP, respectively) [10,11,12,13]. However, in order to understand the mechanisms and patterns of muscle activation during different body positions, it would be ideal to link these assessments to surface electromyography (sEMG). This combination could prove useful in understanding the mechanisms involved in differentiating the activation patterns of different respiratory muscles. Moreover, studying respiratory muscle recruitment and muscle activation during different body positions could be valuable to determine clinical conditions of respiratory muscle weakness. Thus, our study investigates the mechanisms involved in differentiating the activation patterns of various muscles during respiratory pressure assessments in different body positions.

The sEMG of respiratory muscles has proven to be valuable in assessing both the intensity and pattern of their activation. It serves as a diagnostic tool for the detection of neuromuscular disorders, and when combined with respiratory muscle strength tests, also provides insights into the effectiveness of muscle contractility [14]. The simultaneous assessment of multiple muscles, including both primary and accessory respiratory muscles, enhances our understanding of muscle activation patterns and the interactions between them. This comprehensive approach promotes a better understanding of respiratory mechanics and functionality [15].

Given the variability in findings regarding the effects of body position on respiratory muscle strength, this study aims to evaluate the effects of body position on muscle activation patterns during assessments of maximal respiratory pressures by both the oral and nasal routes in healthy adults.

## 2. Materials and Methods

### 2.1. Study Type and Participants

This observational, cross-sectional study was conducted in Natal city, Brazil. This study was reported following the “Strengthening the Reporting of Observational Studies in Epidemiology” (STROBE) statement [16].

Sample healthy individuals were recruited by convenience and invited to participate based on the following inclusion criteria: (1) healthy individuals of both sexes aged between 18 and 29 years with a body mass index between 18.5 and 24.9 kg/m^2^; (2) normal pulmonary function (i.e., forced vital capacity (FVC) greater than 80% of predicted and ratio between FVC and forced expiratory volume in the first second (FVC/FEV1 ratio) greater than 0.7 L or 80% of predicted) [17]; (3) no history of respiratory, neuromuscular or cardiovascular disease; (4) no influenza or colds one week prior to or during the assessment; (5) no use of psychotropic medication or muscle relaxants; and (6) not pregnant. Exclusion criteria were: (1) inability to understand and perform pulmonary function maneuvers or refusal to participate in the study; (2) MIP or MEP less than 80% of predicted values or poor data quality; (3) nasal congestion; and (4) smoker. The Ethics Committee (approval number 3.084.956) approved the present work in accordance with the tenets of the Declaration of Helsinki, and all participants were informed about the objectives and methods of the study and signed the written informed consent form.

### 2.2. Procedures

**Figure 1:**
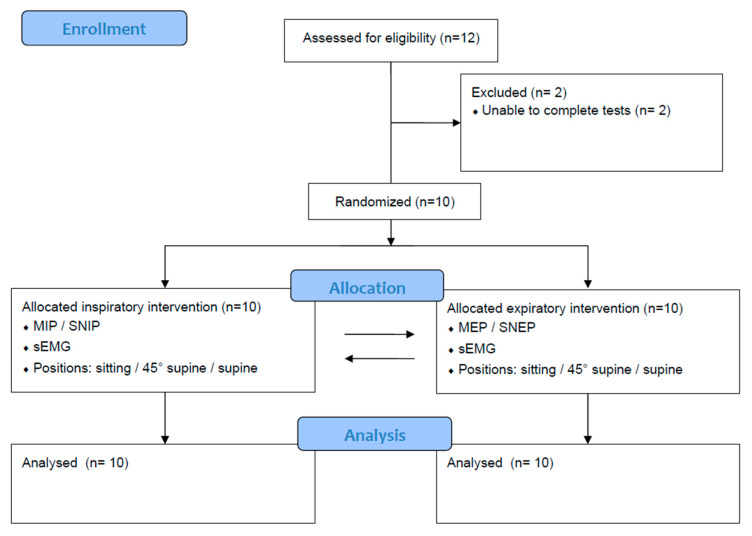
Study flowchart.

Clinical history, anthropometric and lung function data were collected from the participants prior to testing for maximum inspiratory and expiratory pressures at the mouth and nasal tests (MIP, MEP, SNIP and SNEP). The evaluation protocol for each subject spanned two days (session X or session Y) with no predetermined interval between them. Each day consisted of either an inspiratory session with maneuvers to assess inspiratory muscle strength (MIP and SNIP) or an expiratory session to assess expiratory muscle strength (MEP and SNEP). On the first day, participants underwent personal and anthropometric assessments (including weight, height and BMI) and spirometry testing before starting the protocol. Respiratory muscle strength maneuvers (MIP, MEP, SNIP and SNEP) and electromyographic activity (EMG) were performed in three randomized positions: sitting without back support, 45° supine and supine. The type of session (inspiratory or expiratory) and the sequence of positions during the assessments were randomized by simply drawing from opaque envelopes. Figure 1 shows the study flowchart.

#### 2.2.1. Spirometry

Pulmonary function testing was performed using the KoKo DigiDoser^®^ (Longmont, CO, USA) with participants comfortably seated in a chair with a backrest and armrests. A nose clip was used during the procedure to prevent air leakage through the upper airways, according to the American Thoracic Society/European Respiratory Society technical standards [18], and reference values were derived from calculations based on the Brazilian population [17].

#### 2.2.2. Measurement of MIP, MEP, SNIP and SNEP

The assessment of MIP, MEP, SNIP and SNEP were performed with a hand-held digital manovacuometer (NEPEB-LabCare/UFMG, Belo Horizonte, MG, Brazil). During MIP and MEP assessments, the device was connected to a disposable mouthpiece with a small opening (~2 mm) to prevent glottic closure and minimize facial muscle activity. Adherence to the technical guidelines of the European Respiratory Society [9] and the Brazilian Society of Pulmonology and Tisiology [19]. Participants were instructed to perform between three and five MIP and MEP maneuvers, with a minimum of three being considered acceptable and reproducible. To validate the results, the maximum value obtained after tests could not exceed 10% of the three best maneuvers. The results obtained were compared with reference values for the Brazilian population [20].

The SNIP measurement involved the use of a nasal plug attached to one nostril and required participants to make a rapid and maximal inspiratory effort (sniff), which was verbally prompted by the assessor. Criteria for accepting SNIP maneuvers included achieving the highest peak pressure value without leakage, maintaining the duration of the inspiratory effort for up to 500 ms, initiating maneuvers from functional residual capacity, maintaining peak pressure for less than 50 ms, and observing a pressure waveform that showed smooth curves [21,22]. Compliance with the technical criteria of the European Respiratory Society [9] was ensured, with reference values taken from Araújo et al. [10].

The SNEP assessment was performed using an inflatable face mask (dead space approximately 150 mL) with headgear (Vital Signs, South Orange, NJ, USA). The face mask had two openings: one to relieve the pressure generated during the test (2 mm), and another to allow unhindered breathing between and before maneuvers (15 mm). During the SNEP tests, the second opening was connected to a one-way inspiratory valve. The procedure involved performing maximal and rapid expirations (nose blowing), verbally prompted by the assessor, from the volume of functional residual capacity with the mouth closed. The larger opening was manually occluded during each maneuver and reopened immediately afterwards. Participants were given detailed instructions on how to perform the maneuver. Both the SNIP and SNEP tests consisted of twenty maneuvers each. A 30 s interval separated each test, and two familiarization tests were performed.

#### 2.2.3. Surface Electromyographic of Respiratory Muscles

The sEMG was performed following the recommendations of the Surface EMG for the Non-Invasive Assessment of Muscles guidelines (SENIAM) [23]. Myoelectric signal was assessed using the TeleMyo DTS Desk Receiver^®^ electromyograph (Noraxon U.S.A. Inc., Scottsdale, AZ, USA) equipped with four Clinical DTS wireless sensors (Noraxon U.S.A. Inc., Scottsdale, AZ, USA). Signals were sampled at a frequency of 1500 Hz, filtered with a 500 Hz low pass filter, amplified by a factor of 1000, and subjected to a common mode rejection index greater than 120 dB. Data were stored using MR software version 3.8 (Noraxon U.S.A. Inc., Scottsdale, AZ, USA). Muscle assessment was performed unilaterally on the dominant side, focusing on the sternocleidomastoid muscle (SCM), located in the lower third of the distance between the mastoid process and the sternoclavicular joint [24]; the scalene muscle (ECS), located five centimeters from the sternoclavicular joint [25] and two centimeters above this point; the rectus abdominis muscle (RA), anchored four centimeters from the umbilical scar; and the intercostal muscle (IT), assessed in the second intercostal space, three centimeters from the sternum, in the parasternal region [26].

The electromyographic signal was standardized as a percentage of the root mean square (RMS). To calculate the RMS, the signal underwent several processing steps: first, a 20 Hz high-pass filter was applied to eliminate the offset; next, full-wave rectification was performed to convert signal values to absolute values (all positive); then, smoothing was applied using an RMS algorithm and a 50 ms window to eliminate non-reproducible signals; finally, a 30 Hz high-pass Butterworth filter was used to remove any residual cardiac signal. Muscle activation intervals were then selected based on predetermined time intervals and analyzed using MR software version 3.8 (Noraxon U.S.A. Inc., Scottsdale, AZ, USA) to obtain RMS values.

The signals from the SCM, ECS and RA muscles were normalized to the maximum voluntary isometric contraction (MVIC) and maximum voluntary ventilation (MVV) values for the intercostal (IT) muscle. During MVIC, the activation value of each muscle was standardized to 100%, with activations above or below this threshold considered as increases or decreases in electromyographic activity, respectively.

### 2.3. Statistical Analysis

Results were summarized using the mean as a measure of central tendency and the standard deviation as a measure of dispersion. Comparisons between positions were made using generalized estimating equations, with outcome variables treated as dependent variables and positions as independent variables in all models. The identity link function was used, assuming a normal probability distribution and using an unstructured correlation matrix. Post-hoc comparisons were made using the Bonferroni test. Normality of residuals was assessed using the Shapiro–Wilk test. All statistical analyses were performed using GraphPad Prism software version 8.0 for Windows. The power (β) and effect size (ES) were estimated using GPower software version 3.1.9.2 (University of Düsseldorf, Kiel, Germany) and are detailed in the results section of this study.

## 3. Results

Of the initial cohort of 12 people, two were excluded because they were unable to complete all the tests. Consequently, the study comprised 10 participants, equally divided between five males and five females (Figure 1). Table 1 shows the clinical, anthropometric, lung function and respiratory muscle strength characteristics of the sample.

**Figure 2:**
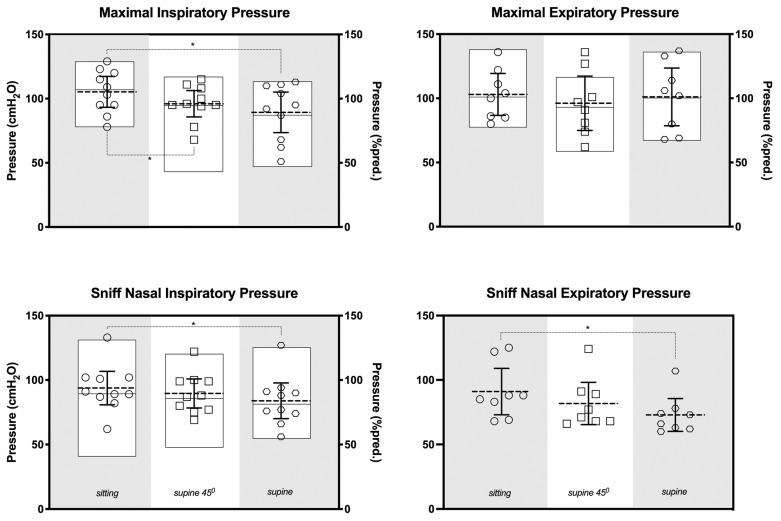
Maximal respiratory mouth and nasal pressures. Symbols represent data in pressure (cmH_2_O); the dotted lines and bars indicate the mean and 95% confidence interval, respectively; the boxes denote the means; maximal and minimal data are expressed as a percentage of predicted normal value (% predicted). * *p* < 0.05 compared to the sitting position.

Figure 2 shows the comparisons of respiratory pressure measurements between the sitting, 45° supine and supine positions, and presents a significantly higher MIP in the sitting position compared to the 45° supine (*p* < 0.05; dz = 3.94; β < 0.99) and supine positions (*p* < 0.05; dz = 2.96; β < 0.99). Additionally, when assessing nasal pressures, statistically significant differences were observed between the sitting and supine positions for both the SNIP (*p* < 0.05; dz = 2.16; β = 0.99) and the SNEP (*p* < 0.05; dz = 3.63; β < 0.99).

**Figure 3:**
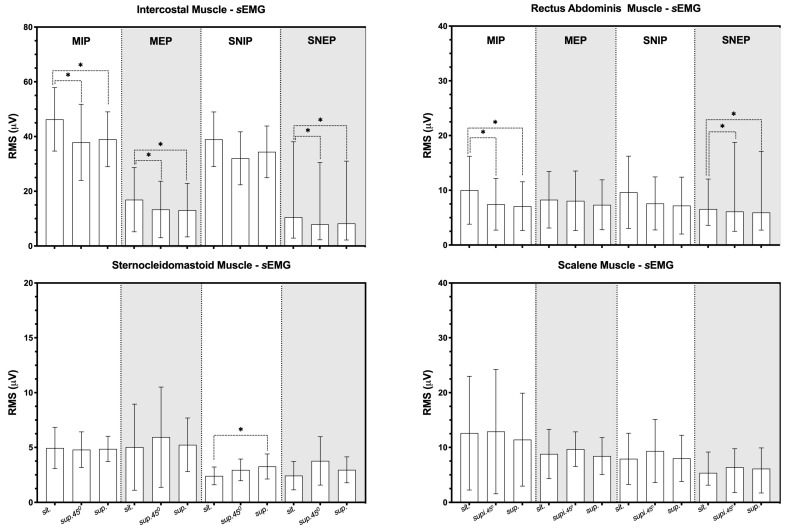
Data presented as mean (95% confidence interval of mean); * *p* < 0.05 compared to the sitting position; RMS: root mean square; sEMG: surface electromyography; MIP: maximal inspiratory pressure; MEP: maximal expiratory pressure; SNIP: sniff nasal inspiratory pressure; SNEP: sniff nasal expiratory pressure.

Figure 3 shows the EMG analyses during the inspiratory and expiratory session. The IT muscle activity in the 45° supine and supine positions decreased compared to in the sitting position during the MIP, MEP and SNEP maneuvers (*p* < 0.05). Furthermore, the RA muscle showed reduced activity in the supine positions at 45° and supine compared to the sitting position during the MIP and SNEP maneuvers (*p* < 0.05). Conversely, there was an increase in SCM muscle activation in the supine position compared to the sitting position during the SNIP maneuver (*p* < 0.05).

Table 2 summarizes the effect size and power test of the study results.

## 4. Discussion

The aim of our study was to evaluate the effects of body position on muscle activation patterns during the maximum inspiratory pressure test generated through both oral and nasal interfaces in healthy adults, specifically analyzing the influence of sitting, 45° supine and supine body positions. The main findings regarding the respiratory pressures were that (1) the MIP was statistically higher in the sitting position compared to the other positions, and (2) the SNIP and SNEP were statistically higher in the sitting position compared to the supine position. The data also suggest that (3) during maximum respiratory pressures generated through the mouth, the activity of the intercostal muscles, on the parasternal area, was statistically higher in the sitting position compared to the other positions. (4) In addition, the activity of the rectus abdominis muscles was also higher in this position during the MIP and SNEP. (5) During nasal route maneuvers, the activation of the sternocleidomastoid muscle was higher in the supine position during the SNIP maneuvers, and (6) the intercostal and rectus abdominis muscles were higher in the sitting position during the SNEP maneuvers.

The results of our study suggest that different muscle activation patterns were observed during maximal oral and nasal interface maneuvers in healthy adults. The sitting position appears to be more effective in generating pressure during MIP, SNIP and SNEP compared to the supine position. Furthermore, during MIP, the sitting position was significantly higher than the 45° supine position. These results add to the existing evidence of muscle effectiveness depending on position and muscle length. The chest wall is characterized by a complex kinematic system, and therefore, muscle activation and pressure generation by the respiratory muscles can vary significantly between different positions [27,28,29,30]. The MIP maneuver involves isometric muscle contraction, so the length–tension relationship of the muscles plays an important role in producing maximal force, particularly in the seated position. In addition, in this position the abdominal muscles help to support the abdominal contents and facilitate effective activation of the diaphragm muscle. In the case of the SNIP and SNEP maneuvers, it is important to consider not only the factors involved in the MIP test, but also the characteristics of these maneuvers. The SNIP and SNEP maneuvers are characterized by being very fast, strong with dynamic contraction, and performed in less than 0.5 s which is considered isotonic time. Muscle length tension also plays an important role.

The electrical activity of the muscles studied showed a very diverse pattern. Intercostal muscle activity contributes to both inspiratory and expiratory tasks in different areas of the thorax, so the higher values observed during MIP and MEP in sitting position are partly supported by the literature. While many previous studies [31,32] have focused on resting breathing rather than maximal effort, making it difficult to determine their role in different positions, our results suggest that the sitting position is more effective in activating of the parasternal intercostal muscles. Regarding the activity of the intercostal muscles, conclusive evidence of their actions is lacking due to the complexity of the three-dimensional structure and function of the rib cage and the breathing process. However, the most widely accepted theory, proposed by Hamberger [33], describes the composition and anatomy of the parasternal muscles, which are distinct from conventional internal intercostal muscles in the rib cage. These muscles are part of the internal intercostal group and are capable of raising the ribs and inflating the lungs. The results obtained are in agreement with the existing literature regarding the effort required to perform strength tests of the inspiratory and expiratory muscles, which are conducted against an occluded airway.

The activation of the rectus abdominis muscles is also significantly higher in the sitting position than in other positions. In a previous study by Costa, Almeida and Ribeiro [34], sixty-three healthy and predominantly female subjects with a mean age of 19.7 ± 1.5 years were studied to evaluate the effects of three different body positions on MIP and MEP. These positions included sitting, supine and semi-upright sitting at a 45° angle; this latter position is very similar to the one used in our study, considering the thorax position. Their results were very similar to those of our study in terms of the maximum inspiratory pressure (MIP) and maximum expiratory pressure (MEP) values observed during sitting compared to the other body positions. However, other studies, such as those by Koulouris et al. [5] and Fiz et al. [35], have not found similar results, even with different methodologies.

Koulouris et al. [5] had different main objectives. These authors studied the influence of abdominal taping on the improvement in transdiaphragmatic pressure induced by phrenic nerve stimulation. The influence of transdiaphragmatic pressure was assessed during the SNIP test in three body positions: seated, semi-supine on a bed with the upper body tilted 30°, and supine. Secondly, MIP and MEP were assessed in these positions in six healthy physiologists (five male, one female, age range 30–45 years). No significant differences were found between the results of the generated pressures. Another study by Fiz et al. [35] using a relatively different methodology, aimed to assess MIP and MEP in fifteen healthy male subjects, with an average age of 27.14 years, in two different positions: sitting and standing. They also found no difference in respiratory muscle strength values between the two positions. Another study from Naitoh et al. [36] aimed to investigate the influence of body position on lung function, including MIP and MEP, and other data related to lung function and chest wall motion. Twenty healthy volunteers (fifteen men and five women) with a mean age of 28.0 ± 1.4 years participated in the study. Measurements were taken in the sitting position and in six supine positions: supine, left retroversion with 45° inclination (LR), left anteversion with 45° inclination (LA), right retroversion with 45° inclination (RR), right anteversion with 45° inclination (RA), and prone position. The authors found no differences in MIP or MEP between the recumbent positions. In line with these results, a brief communication published by Ng and Stokes [3] also did not find any difference in MIP or MEP when measured in half-lying or sitting postures. However, this study was a brief communication and thus lacked detailed information about the data and results. In a more recent study, Albarrati et al. [37] compared the effects of upright and slouched sitting postures on respiratory muscle strength in 35 healthy young males, and SNIP tests were conducted on the participants in both sitting positions. The results demonstrated a significant difference in SNIP scores between two postures, with a mean difference of 8.7 cmH_2_O. The study emphasizes the significance of body posture in influencing respiratory muscle strength and function, demonstrating that the slouched sitting position reduced diaphragm tension and movement. The data presented in the study corroborate the importance of body posture on respiratory muscle strength, which is in line with our results.

Finally, two studies found data that are consistent with the results found in our study: Segizbaeva et al. [38] and Badr et al. [39]. Segizbaeva et al. [38] conducted a study very similar to ours. They assessed the MIP and surface electromyography of various muscles in six positions: standing, sitting, supine, lateral decubitus and head down, with the subject’s body at a 30° angle and the head lower than the feet, involving six men and four women. The results showed that the head-down position resulted in significantly lower MIP values compared to the standing position, with a reduction of 23% in men and 27% in women. In addition, using surface electromyography data, they found that the genioglossus muscles were significantly more active in the head-down and supine positions and less active in the right and left lateral positions compared to the reference standing position. In addition, the parasternal and sternocleidomastoid muscles showed significantly less activity in the head-down position than in the standing position. However, none of the results found by the authors are comparable to ours due to methodological differences between the studies. Badr et al. [39], on 25 adults with normal respiratory function and 11 adults with chronic airflow limitation, studied the effects of seven different body positions: standing, chair sitting, long sitting, three-quarter sitting, supine, side lying and head down, on MEP and peak expiratory flow rate (PEFR). The authors found that body position had a significant effect on MEP and PEFR in subjects in the PEFR group. For MEP, standing gave the highest results (143 ± 10 cmH_2_O), significantly higher than chair sitting. Sitting in a chair showed a significantly higher MEP than all other positions, whereas the head-down position showed a significantly lower MEP than all other positions (108 ± 9 cmH_2_O). For PEFR, the standing position produced the highest rates (571 ± 24 mL/s), significantly higher than all other positions, whereas the head-down position produced significantly lower rates (486 ± 23 mL/s).

The physiological behavior of the respiratory muscles in different body positions has not yet been consolidated in the literature. In addition, our study included the evaluation of new maneuvers in this context. The SNIP and SNEP tests, although similar to the MIP and MEP, are considered to be different tests in terms of their execution from a respiratory muscle physiology perspective. The practical implications of our findings relate to the importance of respiratory muscle activity in several physiological and pathophysiological situations for a more efficient contraction pattern. Situations such as sports performance and assessment and respiratory, cardiac and neuromuscular rehabilitation activities rely heavily on respiratory muscle function. These findings have important implications for exercise physiology and clinical practice in respiratory, cardiac and neuromuscular rehabilitation. They highlight the importance of body position during respiratory maneuvers when measuring or using as a treatment (inspiratory muscle training, breathing techniques, airways clearance techniques, etc.), with the sitting position showing superior performance on MIP, SNIP and SNEP compared to the supine position. This suggests that optimizing body position during respiratory muscle training exercises may enhance the activation of specific muscle groups, such as the intercostal and rectus abdominis muscles during mouth maneuvers and the sternocleidomastoid muscle during nasal maneuvers. Understanding these positional influences can guide the development of targeted exercise protocols to improve respiratory muscle function and optimize rehabilitation outcomes for patients.

Romei et al. [30] studied the kinematics of the chest wall and observed that the breathing pattern was significantly influenced by body position. The more inclined positions caused a reduction in the movement of the rib cage, demonstrating the difficulty of generating greater pressures the more inclined the posture, which suggests a mechanical advantage in more upright postures. 

Limitations of this study include a relatively small sample size, which may limit the generalizability of the findings as well as extrapolation of the results to patients or older individuals with worse muscle function. In addition, the study is small in scope, possibly due to resource or time constraints. However, it is noteworthy that despite these limitations, this study used a reliable methodology. Furthermore, it introduced novel assessments of respiratory muscles, such as SNIP and SNEP, and thus contributed to the advancement of knowledge in this field.

## 5. Conclusions

In conclusion, our study investigated the effect of body position on muscle activation during maximum inspiratory pressure testing in healthy adults, focusing on the sitting, 45° supine and supine positions. The results showed significant differences in inspiratory pressures between positions, with higher values observed in the sitting position compared to the supine position. The sitting posture, in particular, proved to be advantageous by allowing greater activation of the intercostal and rectus abdominis muscles, especially during nasal maneuvers. These findings underscore the critical importance of the sitting posture in respiratory tests, as it appears to optimize respiratory muscle function, providing a more accurate assessment of respiratory muscle strength. Furthermore, the results suggest that the sitting position may have significant implications for respiratory rehabilitation, offering a more effective positioning for respiratory exercises and therapeutic interventions. Despite limitations such as a small sample size and limitations in scope, this study provides valuable insights and introduces novel assessments that advance our understanding of respiratory muscle function in different positions. Further research is warranted to explore these findings and their application in different clinical settings.

## Figures and Tables

**Table 1:** Descriptive statistics of the sample.

Variables	Descriptive Statistic
Age (years)	23.80 ± 2.49
Weight (kg)	64.56 ± 8.29
Height (m)	1.70 ± 0.08
BMI (kg/m²)	22.39 ± 1.62
FVC (L)	4.42 ± 0.63
FVC (% predicted)	98.50 ± 9.19
FEV_1_ (L)	3.70 ± 0.50
FEV_1_ (% predicted)	95.60 ± 8.10
FEV_1_/FVC	0.84 ± 0.06
MIP (cmH_2_O)	105.30 ± 16.78
MIP (% predicted)	90.97 ± 16.43
MEP (cmH_2_O)	103.00 ± 19.52
MEP (% predicted)	68.40 ± 40.11
SNIP (cmH_2_O)	91.80 ± 22.62
SNIP (% predicted)	82.37 ± 21.58
SNEP (cmH_2_O)	91.00 ± 21.54

Data expressed as mean ± standard deviation. BMI: body mass index; FEV_1_: forced expiratory volume in 1 s; FVC: forced vital capacity; MIP: maximal inspiratory pressure; MEP: maximal expiratory pressure; SNIP: nasal inspiratory pressure; SNEP: nasal expiratory pressure.

**Table 2:** Power and effect size.

	Sitting vs. 45° Supine		45° Supine vs. Supine		Sitting vs. Supine	
	Cohen dz	(β)	Cohen dz	(β)	Cohen dz	(β)
Inspiratory session						
MIP	2.96	<0.99	1.28	0.95	3.94	<0.99
SNIP	1.44	0.98	1.72	0.99	2.16	0.99
SCM_MIP	0.48	0.26	0.37	0.18	0.12	0.06
ECS _MIP	0.06	0.05	0.78	0.57	0.84	0.63
RA_MIP	2.04	0.99	0.49	0.27	1.99	0.99
IT_MIP	3.31	<0.99	0.41	0.21	3.38	<0.99
SCM _SNIP	0.91	0.70	1.31	0.94	1.72	0.99
ECS_SNIP	1.26	0.93	1.64	0.99	0.30	0.13
RA_SNIP	1.45	0.98	0.97	0.76	1.61	0.99
IT_SNIP	3.02	<0.99	2.35	0.99	2.40	0,99
Expiratory session						
MEP	1.01	0.81	0.89	0.71	0.29	0.14
SNEP	1.80	0.99	1.46	0.98	3.63	<0.99
SCM _MEP	1.13	0.99	0.4	0.15	0.64	0.42
ECS_MEP	0.08	0.06	0.06	0.05	0.22	0.09
RA_MEP	0.25	0.10	0.64	0.42	0.41	0.2
IT_MEP	3.47	<0.99	0.29	0.13	3.02	<0.99
SCM_SNEP	1.44	0.98	1.42	0.97	1.14	0.87
ECS_SNEP	0.06	0.05	0.41	0.21	0.42	0.21
RA_SNEP	2.36	0.99	0.75	0.54	1.96	0.99
IT_SNEP	2.52	0.99	1.11	0.86	3.37	<0.99

Data presented: Power (β) and effect size for Cohen dz. SCM: sternocleidomastoid muscle; ECS: scalene muscle; MIP: maximal inspiratory pressure; IT: intercostal muscle; RA: rectus abdominis muscle; SNIP: sniff nasal inspiratory pressure; MEP: maximal expiratory pressure; SNEP: sniff nasal expiratory pressure.

## Data Availability

The data can be available upon request to the corresponding author.
